# Complex Leadership in Healthcare: A Scoping Review

**DOI:** 10.15171/ijhpm.2018.75

**Published:** 2018-09-01

**Authors:** Zakaria Belrhiti, Ariadna Nebot Giralt, Bruno Marchal

**Affiliations:** ^1^National School of Public Health, Rabat, Morocco.; ^2^Department of Public Health, Institute of Tropical Medicine, Antwerpen, Belgium.; ^3^Vrije Universiteit Brussel, Brussels, Belgium.

**Keywords:** Complex Leadership, Complexity, Leadership, Healthcare, Scoping Review

## Abstract

**Background:** Nowadays, health systems are generally acknowledged to be complex social systems. Consequently, scholars, academics, practitioners, and policy-makers are exploring how to adopt a complexity perspective in health policy and system research. While leadership and complexity has been studied extensively outside health, the implications of complexity theories for the study of leadership in healthcare have received limited attention. We carried out a scoping review of complex leadership (CL) in healthcare to investigate how CL in healthcare has been defined, theorised and conceptualised and to explore how ‘CL’ has been applied in healthcare settings.

**Methods:** We followed the methodological steps proposed by (Arksey and O’Malley, 2005): (1) specifying the research question, (2) identifying relevant studies, (3) study selection, (4) charting the data, (5) collating and summarizing the findings, and (6) reporting the results. We searched using Medline, Psychinfo, Wiley online library, and Google Scholar. Our inclusion criteria were: publication type (peer reviewed articles, theses, and book chapters); phenomenon of interest: complex leadership; context: healthcare and period of publication: between 2000 and 2016.

**Results:** Our search and selection resulted in 37 papers (16 conceptual papers, 14 empirical studies and 7 advocacy papers). We note that empirical studies on CL are few and almost all research reported by these papers was carried out in the North (mainly in USA and UK). We found that there is some variation in definitions of CL. Furthermore, the research papers adopt mostly an explorative or explanatory approach and do not focus on assessing effectiveness of CL approaches. Finally, we found that the majority of researchers seem to adhere to the mathematical complexity perspective.

**Conclusion:** Complexity concepts derived from natural sciences may not automatically fit management of health services. Further research into how social complexity theories may offer researchers useful grounds to empirically test CL theories in health settings is warranted. Specific attention should be paid to the multi-layered nature of leadership.

## Background

Nowadays, health systems are acknowledged to be complex systems, and often, they are described as messy and unpredictable. Consequently, there is a growing awareness among scholars, academics, practitioners, and policy-makers of the need to adopt a complexity perspective in health policy and system research.^1-3^ Less attention has been paid to the consequences of the complex nature of the health system for management and leadership.

During the 1960s, theory on leadership moved away from the trait and personality theories towards theories that recognised the importance of leadership styles and behaviours.^4^ Contingency leadership, developed by Fiedler^5^ holds that managers have a preferred style of leading, which ranges from task-orientated to relation-orientated styles. Since all leadership styles suit some situations better than others, leaders are more effective in some situations than in others. The resulting situational favourableness to the leader is influenced by, for instance, the nature of the task at hand, the type of staff and the position of the leader in the group. Other authors differentiated between structuring and supportive styles or the structuring and the considering style (see Parry and Bryman^6^ for more details). Situational leadership^7^ is related to contingency leadership. In this view, there is no universalistic ‘best leadership’ approach. Effective leaders adapt their leadership style to the nature of the task, the staff’s capacity and experience with the task and the environment. The approach to leadership in these schools is transactional, ultimately aiming at aligning staff to the organisational goals through task definition, performance assessment, ‘reinforcement’ of positive behaviour and ‘punishment’ of negative behaviour.^8^

During the 1980s, the *transformational* leadership school emerged, according to which effective leaders stimulate their personnel’s awareness of the value of their work and thus trigger the individual’s internal motivation, thereby focusing their attention on organisational goals (and not only personal goals).^8-10^ In practice, transformational leaders do so by being a role model, communicating a clear vision and inspiring staff. This school was based on research of leaders who developed breakthroughs in the US industry, which found that such leaders were charismatic and visionary.^11^ However, the limitations of transformational leadership were quickly identified in terms of the dark side of charisma and toxic or destructive leadership.^12,13^ Since around 2000, complex leadership (CL) has been applied in healthcare management and healthcare organization theory fields.^14^

In this paper, leadership is regarded as a behaviour or set of behaviours that emerges from the interaction among individuals and groups in organizations occurring throughout the whole organisation, and not a role or function formally assigned to an individual (See Plowman and Duchon,^15^ Uhl Bien et al,^16^ and Marion and Uhl-Bien^17^). CL scholars like Uhl-Bien and Marion^16,17^ argue that leadership in complex situations or organisations requires adopting a complexity lens. They call for a transformational, collaborative, reflective and relationship-based leadership style. However, in the field of healthcare, relatively little attention is given to how leaders would best deal with complexity.^18,19^ Notable exceptions include Plsek et al^20^ and Kernick.^21^

In order to investigate how CL in healthcare has been defined, theorised and conceptualised, we carried out a scoping review of CL in healthcare. We present an overview of how CL is discussed in the health literature. We discuss the currently used definitions of CL, the seminal authors and the extent to which CL competencies or practices are discussed in the literature. We end by identifying research gaps and suggest a research agenda.

## Methods

We adopted the guidance for scoping reviews provided by Arksey and O’Malley^22^ and refined by Anderson et al,^23^ Daudt et al,^24^ and Levac et al.^23^ We followed the steps described by Arksey and O’Malley^22^: (1) specifying the research question, (2) identifying relevant studies, (3) study selection, (4) charting the data, (5) collating and summarizing the findings, and (6) reporting the results.

### 1. The Review Question

We defined the review questions as follows:

- How is the notion of ‘CL’ in healthcare defined, theorised and conceptualised?
- How has the concept of ‘CL’ been explored and operationalised in healthcare settings?
- We specifically aimed at:
- mapping key conceptual and operational definitions of CL
- identifying seminal authors and works
- identifying the underlying key complexity traditions (social versus mathematical complexity – see below)
- identifying research gaps and priorities for further research

### 2. Identification of Relevant Studies

### Search Strategy and Sources

We searched four databases (Medline, Psychinfo, Wiley online library and Google Scholar). The search strategies and scope are presented in Table 1. We identified additional sources through manual searching, citation tracking and snowballing from reference lists.

**Table 1:** Search Strategies and Sources

**Sources**	**Date**	**Search Strategy**	**Database Scope**
Psychinfo	15/10/2016	Leadership AND (complex OR Complexity OR Complex adaptive systems OR emergence)	Psychology, book chapters
Medline	15/10/2016	(Complexity leadership[Title/Abstract]) OR Complex) AND ("Leadership"[Mesh] OR leadership)	Public health; health system research
Wiley online library	17/10/2016	Leadership AND (complex OR complexity OR Complex adaptive systems OR emergence)	Organizational, psychology book chapter
Google Scholar	15/10/2016	Leadership AND (complex OR complexity OR Complex adaptive systems OR emergence)	Psychology, Organizational studies book chapters

The scope of the study was adapted iteratively after discussion in the review team in order to balance between feasibility, time constraints and breadth of the scoping study.

### 3. Study Selection

### Inclusion Criteria

We included published papers that explicitly mention ‘complex leadership’ or ‘complexity leadership’ in the publication title or abstract or that mention principles of complexity theory (complex adaptive system [CAS], adaptive leadership, enabling, emergence, non-linearity) in association with ‘leadership’ (See Supplementary file 1).

We defined the inclusion criteria as:

- Publication type: peer reviewed articles, theses and book chapters
- Phenomenon of interest: CL
- Context: healthcare
- Period of publication: between 2000 and 2016

### Exclusion Criteria

We excluded the grey literature, commentaries, conference proceedings and book reviews. Papers discussing only other forms of leadership (transactional, transformational, engaging, distributed, shared or servant leadership) were excluded. All non-health papers are excluded from this review. Studies carried out in non-healthcare settings that might be of interest to other researchers are listed in Supplementary file 2.

### The Search Process

**Figure 1:**
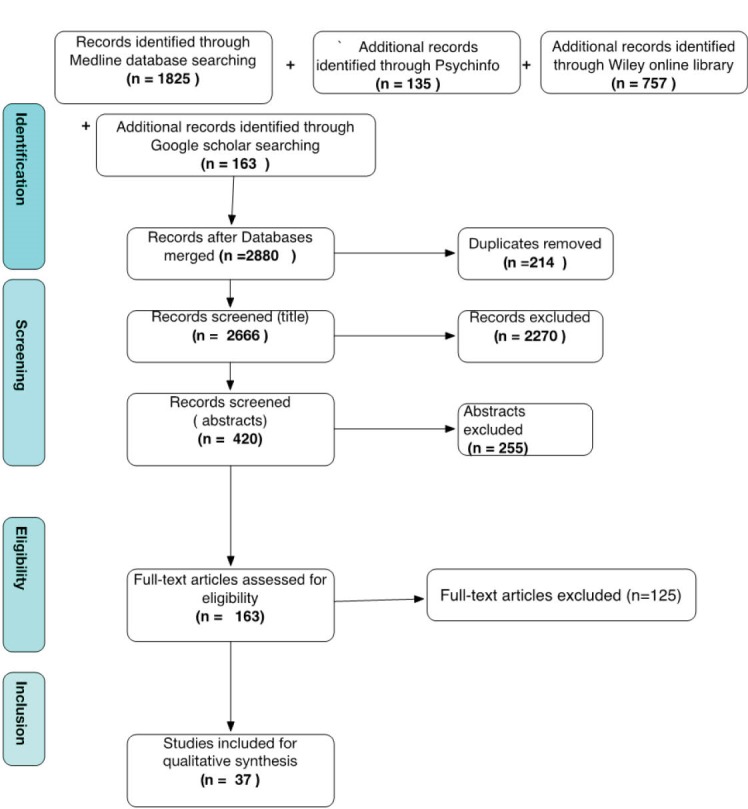
The PRISMA Flow Chart.

Our search and selection resulted in 37 papers (Table 2, Supplementary file 1). Figure 1 summarises the steps of the selection process according the PRISMA statement.^26^ The three authors were involved in the screening process, which was led by the first author. The assessment of inter-rater reliability using the Cohen’s Kappa coefficient (K = 0.675)^ [1]^ showed a good agreement (according to Cooper et al^27^ and Orwin & Vevea^28^) on a random sample of 20% of records using the Random function in the Excel database (Supplementary file 3). Disagreement on 7 references was resolved through discussion and full text screening by the three authors.

**Table 2:** Articles Characteristics, Research Discipline and Fields

**Author, Date**	**Country Origin of Publication**	**Type of Articles**	**Discipline (Research Tradition)**	**Field of the Interests**
(Anderson, 2000)^29^	USA	Conceptual	Nursing research	Interdependence between administrative and non-clinical decisions
(Burns, 2001)^30^	USA	Primary study	Management	Hospital management
(Plsek, 2001)^31^	USA and UK	Advocacy papers	Health service management	Design of healthcare delivery for elderly people (NHS)
(Minas, 2005)^32^	Australia	Conceptual	Public health	Mental health services
(Penprase, 2005)^33^	USA	Conceptual	Research	Nursing leadership
(Forbes-Thompson, 2007)^34^	USA	Primary study	Nursing research	Nursing homes and residential care
(Ford, 2009)^35^	USA	Primary study	Health service management	Hospital management
(Chadwick, 2010)^36^	USA	Primary study	Nursing research	Collaboration physician-nurse in perioperative area
(Davidson, 2010)^37^	USA	Conceptual	Leadership studies	Healthcare leadership
(Gonnering, 2010)^38^	USA	Advocacy papers	Medical education	Clinical practice
(Hanson, 2010)^39^	USA	Primary study	Health service management	Hospital laboratory
(Martin, 2010)^40^	Ireland	Advocacy papers	Communication (sense making)	Medicine, clinical practice and health systems
(Ott, 2010)^41^	USA	Primary study (PhD thesis)	Education	Radical product innovation efforts in biomedical context
(Price, 2011)^42^	UK	Advocacy papers	Medical education	Primary care and family medicine
(Bailey, 2012)^43^	USA	Conceptual	Nursing research	Clinical practice (interaction between practitioner and patient)
(McCarthy, 2012)^44^	UK	Primary study (PhD thesis)	Psychology (positivist perspective)	Healthcare acute hospital
(Sturmberg, 2012)^45^	UK and Canada	Advocacy papers	Psychology	Medicine and healthcare
(Weberg, 2012)^46^	USA	Conceptual	Nursing research	Clinical practice
(Corazzini, 2013)^47^	USA	Primary study	Nursing research	Nursing leadership
(Lindstrom, 2013)^48^	USA	Conceptual	Leadership studies	Leadership in healthcare
(Weberg, 2013)^49^	USA	Primary study (PhD thesis)	Nursing research	Nursing education
(Cohn, 2014)^50^	USA	Conceptual	Leadership studies	Nursing leadership
(Gilson, 2014)^51^	South Africa	Primary study	Health system research	Primary healthcare in South Africa
(Prashanth, 2014)^52^	India	Primary study	Public health (realist perspective)	Capacity building programs
(Viitala, 2014)^53^	Finland	Primary study	Nursing research (social constructivism)	Hospital nursing leadership
(Anderson, 2015)^54^	USA	Conceptual	Nursing research	Chronic non-communicable disease
(Crowell, 2015)^55^	USA	Conceptual	Nursing research	Nursing
(Grady, 2015)^56^	Canada	Primary study (PhD thesis)	Business administration (constructivist perspective)	Physician leadership
(Kwamie, 2015)^57^	Ghana	Primary study	Health system research (realist approach)	Decision making space: district health management teams in Ghana
(Linderman, 2015)^58^	USA	Conceptual	Nursing research	Nursing leadership
(McKimm, 2015)^59^	UK	Conceptual	Change management	Medical field
(Porter-O'Grady, 2015)^60^	USA	Conceptual	Nursing research	Nursing
(Prescott, 2015)^61^	UK	Advocacy papers	Management	National Health System (NHS/UK)
(Arena, 2016)^62^	USA	Advocacy papers	Human resource management	Innovation and adaptation (healthcare system, medical equipment)
(Howard, 2016)^63^	UK	Conceptual	Psychology	Leadership in pharmaceutical industry
(Miller, 2016)^64^	USA	Conceptual	Communication (constructivist perspective)	Mental health
(Weberg, 2016)^65^	USA	Conceptual (book chapter)	Nursing research	Implementation of innovation in healthcare

### 4. Charting the Data

From each paper included in the review, we extracted the data using the form presented in Box 1.

**Box 1. Data Extraction Form**

- Author, date Publication country - origin
- Research aim
- Type of paper
- Research tradition
- Definition of complexity principles
- Conceptual definition of CL
- Main features and practical implication for leadership development
- Underlying theories
- Argument for using complexity theory in leadership
- Arguments against using complexity theory in leadership
- Research gaps and methodological development

## Results

### Overview of the Papers

**Figure 2:**
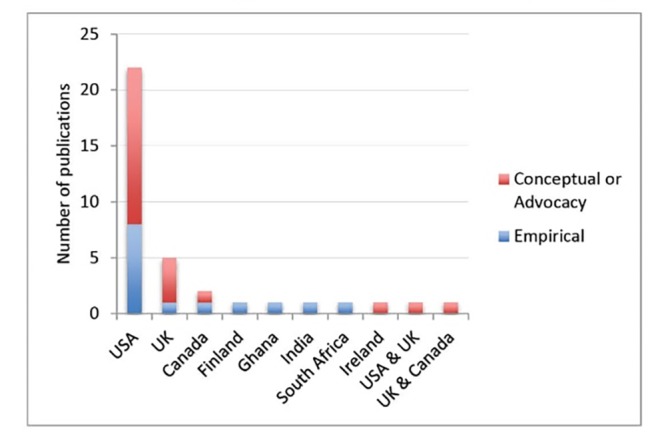
Number, Type of Publications by Country of Origin.

We first present an overview of the papers, addressing the question how CL is being used in the health literature. This review comprises 16 conceptual papers (13 articles and 3 book chapters), 14 empirical studies (4 PhD theses and 10 journal articles) and 7 advocacy papers (Table 2). We note that empirical studies on CL are few and almost all research reported by these papers was carried out in the North (mainly in USA and UK) (11 out of 14). Only three primary studies were carried out in low- and middle-income countries (India, Ghana, and South Africa) (Figure 2). Furthermore, the research papers adopt mostly an explorative or explanatory approach and do not focus on assessing effectiveness of CL approaches. We found that the majority of empirical studies adopted the case study design.^34,47,49,51-53,56,57^

We found that the concept of CL in healthcare is mostly taken up by researchers in the field of nursing (n = 16) (see Table 2).

Finally, researchers framed their research question according to different levels of analysis (Supplementary file 4):

- Micro-level (teams and individuals, care units): 18 papers
- Meso-level (hospital, district): 13 papers
- Macro-level (health system): 2 papers

### Seminal Papers

In order to identify the seminal authors and papers, we assessed the number of citations in the reference list of the 37 papers included in our review. In addition, we also used Web of Science^ [2]^ and Google Scholar. The papers most referred to in this review are Uhl-Bien et al,^66^ Uhl-Bien and Marion,^67^ Plsek and Greenhalgh,^68^ and Zimmerman et al^69^ (Table 3).

**Table 3:** Number of Citations of Seminal Papers

**Seminal Papers**	**This Review**	**Web of Science **	** Google Scholar**
(Weick, 1993)^70^	3	NA	3653
(Stacey, 1992)^71^	5	NA	1823
(Plsek and Greenhalgh, 2001)^68^	7	539	1516
(Uhl-Bien et al, 2007)^66^	7	297	1109
(Dooley, 1997)^72^	5	NA	615
(Plsek and Wilson, 2001)^31^	3	192	570
(Anderson et al, 2003)^73^	3	183	386
(Lichtenstein et al, 2006)^74^	3	NA	300
(Kauffman and Macready, 1995)^75^	3	NA	149
(Uhl-Bien and Marion, 2008)^67^	14	NA	130
(Burns, 2001)^30^	3	16	59
(Zimmerman et al, 1998)^69^	9	NA	43
(Thygeson et al, 2010)^76^	4	12	33

Abbreviation: NA, not available.

### Definitions of Complex Leadership: Heterogeneous Definitions Reflect Different Perspectives

Our analysis shows that there are a number of definitions of CL being used in the literature. The main differences in definition relate to three characteristics: (1) the underlying complexity theories, (2) the definition of the scope (comprehensive or narrow), and (3) the claimed applicability (universal or situational) (Table 4, Supplementary files 5 and 6).

**Table 4:** Main Definitions of Complex Leadership

**Author/Date**	**Definitions**
(Anderson, 2000)^29^	“The task of managing a professional CAS is not to know what is going on and then tell others in the organization what to do. But the task is to create a learning organization.”
(Burns, 2001)^30^	“Leadership that uses complexity principles offers opportunities in the chaotic healthcare environment to focus less on prediction and control and more on fostering relationships and creating conditions in which CASs can evolve to produce creative outcomes.”
(Plsek, 2001)^68^	“Effective organisation and delivery of healthcare does not need detailed targets and specifications, nor should it focus primarily on ‘controlling the process’ or ‘overcoming resistance.’ Rather, those who seek to change an organisation should harness the natural creativity and organising ability of its staff and stakeholders through such principles as generative relationships, minimum specification, the positive use of attractors for change, and a constructive approach to variation in areas of practice where there is only moderate certainty and agreement.”
(Ford, 2009)^35^	“Leader effectiveness depends on the ability to foster conditions that allow for a productive future to emerge.” Three fundamental activities that enable managing turbulence in a non-equilibrium environment: (1) how to foster network construction at the frontline, middle, and top of the organization, (2) how to plant seeds to catalyse emergence from the bottom-up (identify knowledge centres within the organization and encourage these centres to communicate with one another and engage in creative problem-solving), and (3) how to nurture systemic thinking.
(Hanson, 2010)^39^	“CLT examines leadership as a process involving networks of highly interactive, interdependent members leading to collaboration, creativity, innovation, and other outcomes needed for organizational adaptation. Complexity leadership incorporates three types of leadership functions: adaptive, enabling, and administrative (Uhl-Bien et al^66^). From a complexity perspective, there exist both positional and informal leaders fulfilling diverse functions. Formal leaders carry the authority of position; informal leaders emerge based on relationship.”
(Viitala, 2014)^53^	“Leadership is seen here as a socially constructed product, which is at the same time institutionalised both in organisations and in a society and also continually being reproduced in everyday situations in communities. (…) The core of the issue is communication, influence and interaction between people and in this process both power and resistance play important role.”
(Weberg, 2016)^65^	“CLT includes leadership recognition of interrelationships, emergence, and fostering innovation, ‘multilevel leadership (administrative, enabling, adaptive)’; a complex interaction of leadership behaviours by multiple individuals in response to emergent opportunities in the internal and the external environments (not a single individual characteristics) that leads to change adaptation and innovation.”

Abbreviations: CAS, complex adaptive system; CLT, Complex Leadership Theory.

### The Underlying Complexity Theories

**Figure 3:**
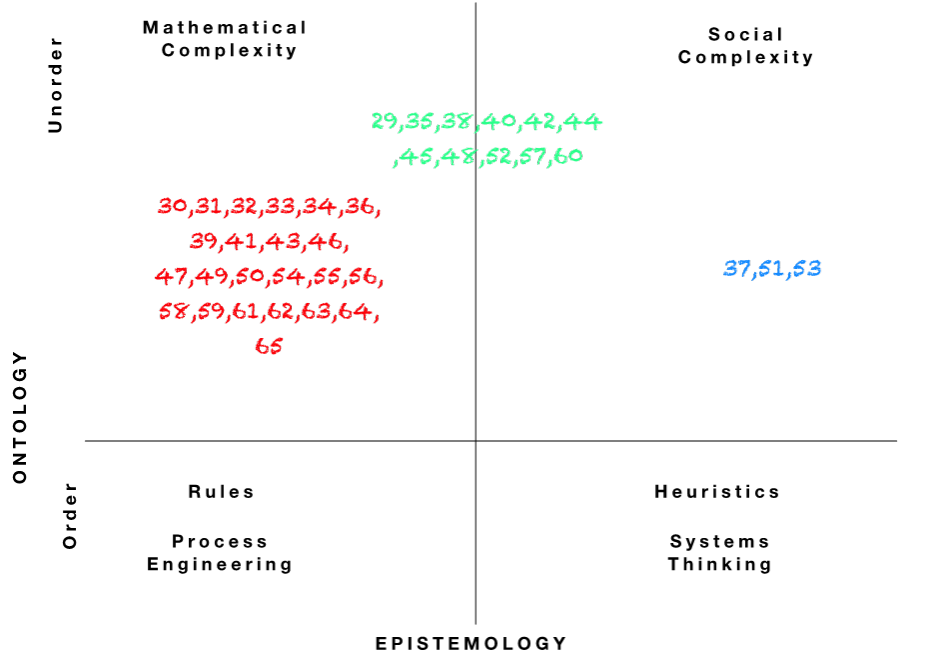
Mapping the Authors in the Landscape of Management Framework.^77^

We used the ‘landscape of management’ framework of Snowden and Stanbridge^77^ to classify the papers included in this review in terms of the complexity perspective they adhere to (Figure 3).

We found that most researchers subscribe to the mathematical complexity perspective. According to Snowden and Stanbridge, the mathematical complexity perspective asserts that the world is unordered and that human behaviour emerges from simple rules or minimum specification. In ordered systems, managers can determine the desired end state, assess the initial situation and consequently set out a series of actions to reach the desired end state. In unordered systems, one cannot do so because of the uncertainty related to how the end state can be attained. Instead, managers set out simple rules that guide the personnel regarding the desired end states and allow them to decide and implement actions locally. Trial and testing allows to learn in a systematic way and to optimise the activities.^77^

Many other scholars we identified refer to the definition of CL by Uhl-Bien et al^66^ (see for instance^39,41,42,44,46,49,56-58,62,64,65^).

The seminal authors we identified can all be classified under the mathematical complexity perspective. They all refer explicitly to concepts of CASs theory. For example, Plsek and Wilson draw upon CAS terminology to explain certain aspects of CL:

*“…effective organisation and delivery of healthcare does not need detailed targets and specifications, nor should it focus primarily on ‘controlling the process’ or ‘overcoming resistance.’ Rather, those who seek to change an organisation should harness the natural creativity and organising ability of its staff and stakeholders through such principles as generative relationships, minimum specification, the positive use of attractors for change, and a constructive approach to variation in areas of practice where there is only moderate certainty and agreement.”* ^31^

The social complexity perspective acknowledges ‘un-order’ and emergence, but considers that this results from the uniqueness of human beings and that it cannot be reduced to simple rules. In this view, humans decide on the basis of social interactions and patterns of past experience. Researchers who adhere to this perspective emphasize the importance of conversation and socially constructed meanings. Authors refer, for instance, to complex responsive systems theory^71^ and critical realism.^78,79^ They focus on meanings and sense making. In this perspective, CL is regarded as a communication process that is socially constructed by the interaction of agents.^51,79^ Viitala suggests the following definition of leadership:

*“Leadership is seen here as a socially constructed product, which is at the same time institutionalised both in organisations and in a society and also continually being reproduced in everyday situations in communities. (…) The core of the issue is communication, influence and interaction between people and in this process both power and resistance play important role.”* ^53^

Our analysis shows that only few authors adopt a social complexity perspective. Gilson, for instance, emphasise the role of leaders in terms of making sense of reality using a complexity lens.^51^

We also noticed that a number of authors seem to combine both perspectives (mathematical and social complexity), for instance Porter-O’Grady^60^ and Prashanth et al.^52^ This is what Snowden and Stanbridge^77^ labelled the *contextual complexity* perspective, arguing that people (ie, managers and researchers) are able to shift between the mathematical and social complexity perspective. Through such multi-ontology sense making, managers or researchers adopt different “*diagnostic techniques, different intervention devices and different forms of measurement depending on the ontological state*.”^77^

### The Definition of the Scope

Definitions of CL can be considered to be comprehensive or narrow. Comprehensive definitions present a multilevel perspective of leadership that is situated at all hierarchical levels of an organisation (top, middle, and line management). The most comprehensive definition is proposed by Uhl-Bien et al,^16^ who present a holistic view of leadership that comprises an administrative, enabling and adaptive dimension of leadership. Their complexity leadership theory (CLT) explores how order emerges from the interactions among agents.^16,67^

*“Adaptive leadership is an emergent, interactive dynamic that is the primary source by which adaptive outcomes are produced in a firm. Administrative leadership is the actions of individuals and groups in formal managerial roles who plan and coordinate organizational activities (the bureaucratic function). Enabling leadership serves to enable (catalyse) adaptive dynamics and help to manage the entanglement between administrative and adaptive leadership (by fostering enabling conditions and managing the innovation-to-organization interface). These roles are entangled within and across people and actions.”* ^16^

Similarly, authors such as Weick 2007^80^ consider that leadership can be located anywhere in the organisation (“constellation leadership”). This view emphasizes that diffused power is beneficial in complex organisations.

In contrast, authors who present a narrow definition of CL locate leadership at the operational level.

*“Leadership emerges in day to day work as people interact with each other to do their jobs. Adaptive leadership is the work that practitioners do to mobilize and support patients to do the adaptive work. Adaptive leadership is fundamentally a non-linear, iterative, reciprocal interaction between the healthcare practitioner and the patient.”* ^43^

**Figure 4:**
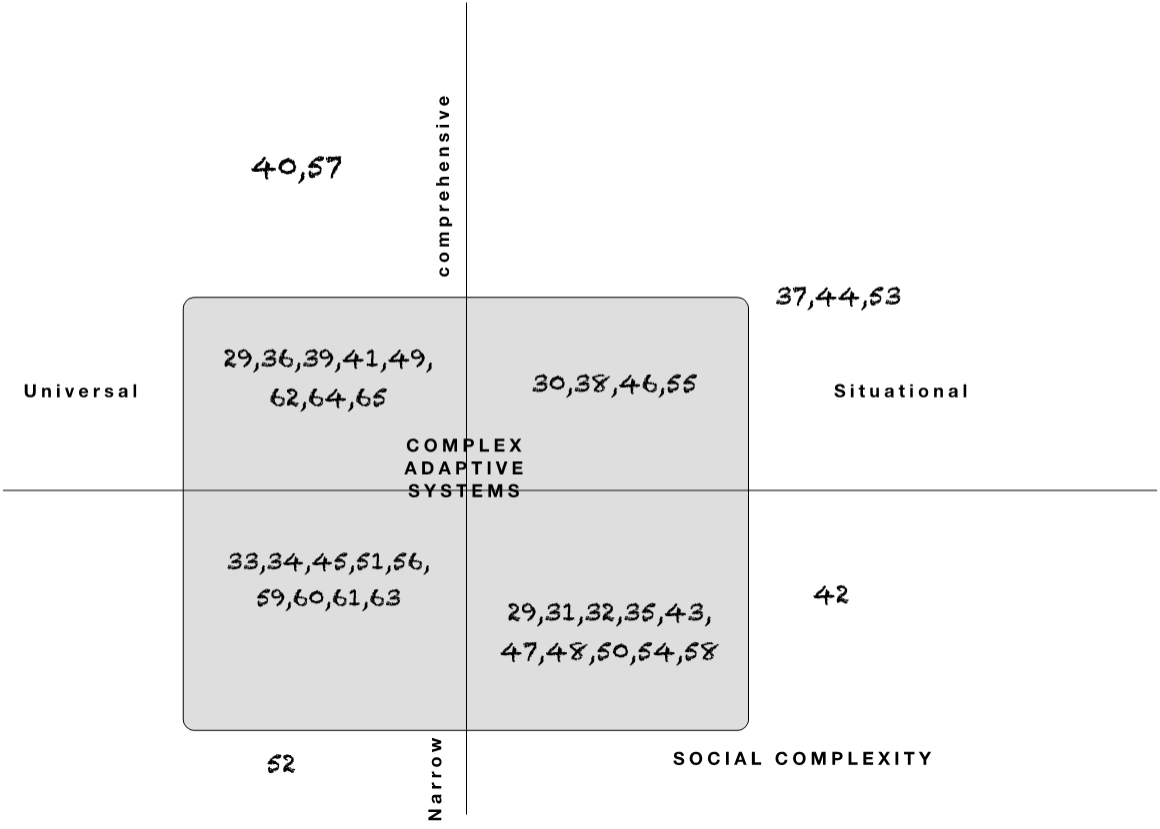
Position of the Papers Along the ‘Complexity Theories,’ ‘Scope,’ and ‘Applicability’ Axes.

Other authors use similar narrow definitions of CL^29,32,34,35,41,43,45,48,56,58-61,63^ (see Figure 4).

### Applicability: Universal Versus Situational Perspective

The definitions of CL can be categorised as universal or situational. Authors adhering to the universal perspective argue that CLT can or should be applied to any situation.^29,33,35,36,39-41,49,51,52,56^

*“There is need for leadership at all levels and in all professions in the complex worlds of NHS institutions.”* ^61^

Their main argument is that healthcare organisations should be considered as CAS: they are characterised by non-linear dynamics, sensitivity to initial conditions, unpredictability of both social behaviours and contextual components, interconnectedness, interdependency and emergence. For these authors, it thus makes sense for leaders in the health system to always apply CL.

*“Organizations embedded with various properties and mechanisms that contribute to collective adaptive capacities and tendencies are described as CASs.”* ^39^

Other scholars suggest that CL should be used in only *complex* situations.^20,30,32,37,42,44,47,48,50,53-55^ The latter are defined as situations or contexts in which the cause-effect relationships are unknown. In such case, leaders should stimulate self-management and support decentralised decision-making. Given that the situation is defined as complex because of the uncertainty related to the root causes of the problems, and thus of the solutions, leaders encourage testing solutions and continuous learning. They set boundaries but do not steer the process.^35^ In this view, simple and complicated events do not require CL; transactional and transformational leadership will be more effective. This view implies that health managers should a priori assess the situation or context, categorize it as simple, complicated or complex, and accordingly apply the most appropriate leadership approach.

*“A new type of leadership is needed within healthcare organizations, based on adaptive capacity, understanding the external environment and connecting with the internal organizational culture and thriving in situations where groups need to learn their way out of unpredictable problems.”* ^46^

In Figure 4, we present how the papers are located against the complexity, scope and applicability axes. It shows that while there is a homogeneous representation across narrow and comprehensive scope, and universal and situation perspective, most authors refer to mathematical complexity.

### Complex Leadership Competencies

We found that only a few authors have described specific competencies or practices related to CL. According to Ford,^35^ complex leaders should be able to:

foster network construction at the frontline, middle and top of the organization,

catalyse emergence from the bottom-up by identifying the knowledge centres within the organization and encouraging these centres to communicate with one another and engage in creative problem-solving, and

nurture systemic thinking.

According to Anderson and McDaniel, “*managers who focus on relationship building, loose coupling, complicating, diversifying, sense making, learning, improvising, and new ways of thinking about the future will be able to create new levers for positive movement in their organizations.”*^29^

We present in Table 5 a set of complexity leadership behaviours in healthcare.

**Table 5:** Set of Complex Leadership Behaviours in Healthcare

**Set of Complex Leadership Behaviours in Healthcare**
1) Adopting and fostering teams to adopt complexity lenses^30,35,36,38,52,55-59^
2) Build a good-enough vision and provide minimum specifications^30-33,55,65^
3) In uncertain situation leading from the edge, using both “clockware” (mechanistic) and “swarmware” (embrace complexity)^30^
4) Tuning your place to the edge by fostering the right degree of information flow, diversity and difference, connections inside and outside the organization, power differential and anxiety^30-32,55,58,65^
5) Deciphering trends and work with paradox and tension^30,33^
6) Listening to the “shadow system.” Informal relationships, gossip, rumor, and hallway conversations that influence people’s mental models and subsequent actions^30,55^
7) Growing complex systems by “chunking,” or allowing them to emerge out of the links among simple systems that work well and are capable of operating independently^30,46^
8) Balancing cooperation and competition^30,36,55^
9) Managing generative relationships by fostering interaction^29,31,32,37,50,51,54,58^
10) Using of interpersonal management tactics (eg, maintaining constructive dialog) to assist in resolving professional issues^29,59^
11) Utilizing loose coupling and weak ties as a strategy for dealing with the dynamic nonlinear nature of the system^29^
12) Planting seeds of emergence by Identifying Knowledge centres (expertise) and value systems of the professional community^29,35,55,65^
13) Developing complicated sets of information-driven networks (frontline/middle/top)^29,35,46,65^
14) Nurturing professional value systems that serve as stabilizers^29,38,49,81^
15) Sense making (reflecting and enhancing awareness about work and contextual conditions)^29,35-37,46,51,54,57-59^
16) Understanding "strange" Attractors (experiences or forces that attract engagement and energies) for change rather than battling resistance and careful sharing of information^31-33,51,55,58,59^
17) Enable liberating structure and foster Learning for capability by assisting staff to accept and adjust to change generate new knowledge, and better performance (eg, lifelong learning, learning networks, Action Learning cycle)^31-33,47,49,53,54,58,65^
18) Articulating values that underpin everything else in the systems^31,32^
19) Providing less answers and less direction and more facilitation creating the conditions in which followers’ behaviours can work through inherent tensions and produce structure and innovation^35^
20) Plant seeds to catalyse emergence from the bottom-up; by identifying knowledge centers within the organization and connecting them for creative problem solving and collective action^35^
21) Accept surprise and embrace unpredictability, Become comfortable with uncomfortable situations^31-33,36,51,57,58^
22) Understand how the people leaders serve are motivated so that interactions can be tailored to ultimately result in quality patient care^36^
23) Meeting the need of patients, staff that roll up into organization mission: bringing in to life the mission and vision of the organization^36^
24) Carry out creative destruction by dismantling rigid systems that allow little variety and are less responsive to their environment^36^
25) Discerning the truth as we engage in the complex responsive processes of relating to one another^37^
26) Stimulating Creative problem solving, practicing mindfulness being openminded and curious^37,58^
27) Being fully aware of our surroundings in the living present particularly to the quality and nature of our interactions and relationships with others^37^
28) Being self-reflective and learn from our mistake and risk taking^37,58,59,65^
29) Shifting from the macro time frame (past, present, future) to the micro time frame (here and now)^49,58^
30) Coevolving, developing larger ecosystems that connect people and their actions across boundaries through seeing and acting from the whole^37,55,56,59,65^
31) Adopt a situational approach in dealing with simple, complicated and complex problems ( tool such as Stacey diagram and plots certainty/agreement are helpful^53,55^
32) Spending as much time advancing the culture "reculturing" of the organization as in strategy implementation^36,59^
33) Leveraging opportunities and suggest alternatives^47,55,56,58,65^
34) Creating conditions for change and adopting a positive deviance approach to change discovering those individual achieving better outcomes, determine what specific behaviours are associated with the better outcomes and then choose to adopt these behaviours^50,59^
35) Promote a collective perspective of leadership^53,56,58^
36) Valuing the importance of middle managers/professional communities personal values^35^
37) Controlling from bottom up and fostering self organization^55^

## Discussion

This review shows that there are relatively little empirical applications of CL in healthcare settings. Virtually all empirical studies have been carried out in the North and focused on exploratory or explanatory research objectives, which reflects other reviews’ findings.^82,83^

We found that there is a wide variation in definitions of CL, even if there are clearly seminal papers. We identified some common themes. First, leadership is increasingly seen as a process of process and less as a process centred on individuals. Second, CL is about fostering interactions and enabling conditions for the emergence of creative behaviours.^84^ Third, CL is associated with positive outcomes such as contributing to learning organisations, creativity, innovation and adaptability. The heterogeneity of CL definitions explains the variety in CL research, but also raises questions related to the generalisability of the concept.

In summary, CL could be defined as a multilevel process throughout the whole organization, as opposed to an individual’s attribute. It is less focused on predicting and controlling the future and more about facilitating staff interaction. It emphasises roles of distributed leadership and learning adaptability. CL fits situations of complex healthcare issues (eg, patients centred care) where there is low certainty and agreement. It is a socially constructed process that includes communication, influence, interaction between individual agents on a day-to-day basis and considers the role of power and resistance. In such situations, leaders stimulate sense making and self-reflection among staff to help them develop new insights into how to deal with the issues at hand (eg, improving quality of care).

We found that most authors may be classified as adhering to the mathematical perspective on complexity, which reflects commentaries of Polack et al,^82^ Burnes^85^ and McKelvey,^86^ who argue that there is an increased application of mathematical complexity in organisational studies in health.

### The Use of Metaphors

In our review, we found very few empirical papers and these present explorative research rather than evidence on effectiveness of CL approaches. This, too, is consistent with findings from other reviews of complexity in health system research^18,87^ and management and organisational studies.^82,85^ It seems that at this stage, scholars on leadership mainly apply complexity in leadership on theoretical and metaphorical grounds rather than on the basis of empirical studies and evidence. We agree with Anderson et al^88^ that there is a need for developing middle range theories on CL and testing them in empirical studies in a variety of settings.

### Can Complex Adaptive System Concepts Be Transposed to Leadership?

Related to the previous point, our review shows that many authors draw concepts from CASs terminology. For instance, Forbes-Thompson et al^34^ and Minas^32^ argue that CL consists of setting simple rules that allow emergent behaviour to happen, the way flocks of birds adopt flight patterns. However, the papers often provide little justification for the fit of CAS concepts to the social world and thus it is not clear whether and exactly how these concepts can be applied to understanding leadership in healthcare organizations. This is similar to the use of CAS concepts in other disciplines. Scholars often take for granted the assumption that organisations can be assimilated in all their aspects to CAS.^14,89^ Such analogy allows them to explain social change as an interaction between agents, groups, and institutions that are operating at different levels. However, Mowles^90^ and other authors argued that complexity concepts derived from natural science may not automatically fit management^86,91-93^ and social settings. The study of social complexity should be rooted in social theory relevant to organizations.^91,94-96^ If not, there is a risk of scientific reductionism.

### Situational or Universal Complex Leadership?

Our analysis also indicated that there is little consensus on when or in which situation CL should be applied. For one set of authors, the complex nature of health systems requires leaders always to apply a complexity perspective. Authors including Uhl-Bien et al,^16^ Hanson and Ford^35,39^ argue that in the current knowledge era, traditional leadership and management approaches are no longer sufficient to deal with the organisational and contextual complexity. Thus, it is argued, context plays a key role in shaping leadership^24,41,46,97,98^ and because of the complex nature of health systems, leaders should always adopt a CL perspective.

Other authors advocate for a more situational approach, arguing that the leadership approach should be used only in complex settings. This approach fits well with sense-making frameworks, such as the Cynefin framework,^99^ the ‘simple-complicated-complex’ frame of Stacey,^71^ Glouberman and Zimmerman^100^ or Stacey’s diagramme.^92^ Here again, the empirical evidence is poorly developed.

### Leadership Effectiveness

Our review showed that the relation between CL and organisational performance is little developed. A number of scholars argue that complex leaders foster interconnectedness, open communication, relationship building, and non-linear processes, and that this contributes to positive outcomes such as collaborative learning, innovation, perceived team performance, and organisational change.^16,57,62,67,74,101,102^ These writers emphasize the need to pay closer attention to the quality and the nature of leadership processes in exploring leadership effectiveness.

Howard, Grady and Weberg examined the abilities needed to improve resilience and trust among healthcare teams.^49,56,63^ Nursing researchers emphasized the need to explore the relationship between CL and specific health outcomes.^46^ Others stress that CL is about interaction among agents. Authors like Marion and Uhl Bien^39,41,44,49,57,62,65,103^ conceive leadership as rooted in the interaction between agents. Understanding these interactions or ‘the space between’ the actors is then a relevant means of investigating the mechanisms that enable processes of adaptation and creation.^66,74,104-107^

### Research Gaps

The papers we reviewed suggest some gaps in research, both in terms of substance and methods.

Content-wise, some authors call for exploring the nature of network dynamics associated with the transformation process, generation of innovation, emergence and diffusion,^108^ shared leadership and organisational adaptability.^109^ Authors like Clancy et al,^110^ Weberg,^46^ and Carter et al^111^ call for less emphasis on computational modelling and simulation. We suggest that leadership scholars should empirically test CL theories in social settings rather than merely use complexity concepts as explanatory metaphors. Further attention should be paid to CL effectiveness on learning, innovation, adaptability and followers’ behaviours. We suggest also that scholars should pay attention to related concepts, such organisational learning and organisational culture theories to build detailed middle range theories.

In terms of research methodology, some stress the need for context-sensitive methods, which should enable identifying the context factors and mechanisms that explain leadership and patterns of behaviours in organisations.^84,112^ They call for exploring how mechanisms, understood as patterns of social interaction, produce specific outcomes, thereby opening the black box of CL effectiveness. Research methods should take into account the multi-layered aspect of leadership and the dynamic interactions over time between context (eg, health policy) and organisational characteristics (power, intentions, codes, organisational culture, followers’ behaviour and expectations…). Others point to the need for rigorous methodologies to study patterns of leadership interaction over time.^32,74,113,114^ Viitala^53^ suggests using ethnography, longitudinal designs and embracing a social constructionist perspective. We would argue that other interesting methodological avenues include case based methodologies (including qualitative comparative analysis^115,116^), the sociology and complexity science toolkit (SACS),^117^ cluster analysis and social network analysis. In general, more empirical research, and particularly in low- and middle-income countries, would enable producing better insights into what constitutes CL and its relation to organisational effectiveness. It would also add contextual validity to concepts mainly developed in the North.

We acknowledge the limitations that are specific to the scoping methodology (for instance, the absence of quality appraisal, and the potential interpretation bias).^22-25,118,119^ We also had to balance comprehensiveness with feasibility. Finally, our search strategy (Table 1) may have overlooked some relevant studies. However, our primary objective was to explore the application of ‘CL’ in health and to contribute to shaping the research agenda and to these ends, the scoping review proved appropriate.

## Conclusion

This review showed how the limited attention in the current literature to applications of CL in healthcare settings. While we identified a number of seminal papers, the definitions of CL are heterogeneous. We found that the majority of researchers seem to adhere to a mathematical complexity perspective. At this stage, there is very little empirical research, while we need a better understanding of the key characteristics of CL and how complex leaders contribute to better healthcare. Although complexity science has been extensively used elsewhere, it is still not much applied in health systems. Further research could focus on how a social complexity perspective could be applied to leadership in healthcare.

## Acknowledgements

We would like to thank Mouloud Benabbou head librarian at the National School of Public Health and Dirk Schoonbaert head librarian at ITM and their teams for their help in the extraction of articles.

We would like to thank the editor and the anonymous reviewers for their insightful comments and Dr. Issam Bennis for reviewing an earlier version of the manuscript.

## Ethical issues

Not applicable.

## Competing interests

Authors declare that they have no competing interests.

## Authors’ contributions

All authors participated in the design of the study and the review process. ZB led the review process and drafted the first manuscript. AN contributed to the selection process, the synthesis process and the revision of the manuscript. BM contributed to the study design, the synthesis process and the drafting of the manuscript. All authors read and approved the final manuscript.

## Authors’ affiliations

^1^National School of Public Health, Rabat, Morocco. ^2^Department of Public Health, Institute of Tropical Medicine, Antwerpen, Belgium. ^3^Vrije Universiteit Brussel, Brussels, Belgium.

## Endnotes


[1] Kappa Coefficient interrater reliability measures the agreement between two authors making simple inclusion/exclusion decisions) and scores as follows: 0.40 to 0.5: fair agreement; 0.60 to 0.7: good agreement, 0.75 and more: excellent agreement.

[2] Until recently, the Web of Science index did not include chapter books nor papers from the field of organisational studies.


## Supplementary files


Supplementary file 1. List of Included Studies.

Supplementary file 2. Complex Leadership Studies in Non-healthcare Settings and Reasons for Exclusion.

Supplementary file 3. Measurement and Interpretation of Kappa Coefficient

Supplementary file 4. Research Question in Complex Leadership

Supplementary file 5. Scope, Epistemology, Theory and Conceptual Definitions of Complex Leadership.

Supplementary file 6. Categorization Criteria of Included Papers.
