# Supplementary material for: Complex Leadership in Healthcare: A Scoping Review
Source: Int J Health Policy Manag. 2018 Sep 1;7(12):1073–84. doi: 10.15171/ijhpm.2018.75 (PMC6358662; doi:10.15171/ijhpm.2018.75)
Supplement: Supplementary file 1 — List of Included Studies. [file ijhpm-7-1073-s001.pdf]

Supplementary file 1. List of Included Studies

Anderson RA, Bailey DE, Jr., Wu B, Corazzini K, McConnell ES, Thygeson NM, Docherty SL: Adaptive leadership framework for chronic illness: framing a research agenda for transforming care delivery. ANS Adv Nurs Sci 2015, 38(2):83-95.

Anderson RA, McDaniel RR, Jr.: Managing health care organizations: where professionalism meets complexity science. Health Care Manage Rev 2000, 25(1):83-92.

Arena MJ, Uhl-Bien M: Complexity Leadership Theory: Shifting from Human Capital to Social Capital. People and Strategy 2016.

Bailey DE, Jr., Docherty SL, Adams JA, Carthron DL, Corazzini K, Day JR, Neglia E, Thygeson M, Anderson RA: Studying the clinical encounter with the Adaptive Leadership framework. J Healthc Leadersh 2012, 2012(4).

Burns JP: Complexity science and leadership in healthcare. J Nurs Adm 2001, 31(10):474-482.

Chadwick MM: Creating order out of chaos: a leadership approach. Aorn j 2010, 91(1):154-170.

Cohn J: Leading Healthcare in Complexity Nursing leadership (Toronto, Ont) 2014, 27(4):52-64.

Corazzini KN, Anderson RA, Day L, McConnell ES, Mueller C, McKinney SH: When a situation is "not black or white": Using adaptive leadership to address complex challenges in nursing home care. Director 2013, 21(4):34-37.

Crowell DM: Complexity Leadership: Nursing's Role in Health-Care Delivery; 2015.

Davidson SJ: Complex responsive processes: a new lens for leadership in twenty-first-century health care. Nurs Forum 2010, 45(2):108-117.

Forbes-Thompson S, Leiker T, Bleich MR: High-performing and low-performing nursing homes: a view from complexity science. Health Care Manage Rev 2007, 32(4):341-351.

Ford R: Complex leadership competency in health care: towards framing a theory of practice. Health Serv Manage Res 2009, 22(3):101-114.

Gilson L, Elloker S, Olckers P, Lehmann U: Advancing the application of systems thinking in health: South African examples of a leadership of sensemaking for primary health care. Health Res Policy Syst 2014, 12:30.

Gonnering RS: Future demands complex leadership. Physician Exec 2010, 36(2):6-10.

Grady CM: Exploring Physician Leadership Development in Health-Care Organizations Through the Lens of Complexity Science. 2015.

Hanson WR, Ford R: Complexity leadership in healthcare: Leader network awareness. Procedia-Social and Behavioral Sciences 2010.

Howarde H: The Influence of Leadership Paradigms and Styles on Pharmaceutical Innovation. In: Value Creation in the Pharmaceutical Industry. edn. Edited by Schuhmacher A, Hinder M, Gassmann O: Wiley-VCH Verlag GmbH & Co. KGaA; 2016: 416-447.

Kwamie A, Agyepong IA, van Dijk H: What Governs District Manager Decision Making? A Case Study of Complex Leadership in Dangme West District, Ghana. Health Systems \& Reform 2015.

Linderman A, Pesut D, Disch J: Sense Making and Knowledge Transfer: Capturing the Knowledge and Wisdom of Nursing Leaders. J Prof Nurs 2015, 31(4):290-297.

Lindstrom RR: Leadership needs to shift in the health system: three emerging perspectives to inform our way forward. Healthc Pap 2013, 13(1):48-54; discussion 78-82.

Martin CM: Making sense of polarities in health organizations for policy and leadership. J Eval Clin Pract 2010, 16(5):990-993.

McCarthy I: The complexity of leadership and organisations. 2012.

McKimm J, Till A: Clinical leadership effectiveness, change and complexity. Br J Hosp Med (Lond) 2015, 76(4):239-243.

Miller CP: Mental health leadership and complexity. Journal of Complexity in Leadership and Management Vol 3 Issue 1/2,:155-161 2016.

Minas H: Leadership for change in complex systems. Australas Psychiatry 2005, 13(1):33-39.

Ott Ann Marie Noller: Through the looking glass of complexity leadership theory: A biomedical case study in radical innovation leadership. 2010.

Penprase B, Norris D: What nurse leaders should know about complex adaptive systems theory. Nurs Leadersh Forum 2005, 9(3):127-132.

Plsek PE, Wilson T: Complexity, leadership, and management in healthcare organisations. Bmj 2001, 323(7315):746-749.

Porter-O'Grady T: Confluence and convergence: team effectiveness in complex systems. Nurs Adm Q 2015, 39(1):78-83.

Prashanth NS, Marchal B, Devadasan N, Kegels G, Criel B: Advancing the application of systems thinking in health: a realist evaluation of a capacity building programme for district managers in Tumkur, India. Health Res Policy Syst 2014, 12:42.

Prescott D, Rowe M: Leadership in systems, organizations and cultures. Br J Hosp Med (Lond) 2015, 76(2):101-104.

Price J: Complexity, Leadership and Management in Primary Care. PrimaryCare 2011.

Sturmberg JP, Martin CM: Leadership and transitions: maintaining the science in complexity and complex systems. J Eval Clin Pract 2012, 18(1):186-189.

Viitala R: Leadership in transformation: a longitudinal study in a nursing organization. J Health Organ Manag 2014, 28(5):602-618.

Weberg D: Complexity leadership: a healthcare imperative. Nurs Forum 2012, 47(4):268-277.

Weberg D: Innovation Leadership Behaviors: Starting the Complexity Journey. In: Leadership for Evidence-Based Innovation in Nursing and Health Professions. edn. Edited by Publishers JaB; 2016.

Weberg DR: Complexity leadership theory and innovation: A new framework for innovation leadership. 2013.
