# Supplementary material for: Complex Leadership in Healthcare: A Scoping Review
Source: Int J Health Policy Manag. 2018 Sep 1;7(12):1073–84. doi: 10.15171/ijhpm.2018.75 (PMC6358662; doi:10.15171/ijhpm.2018.75)
Supplement: Supplementary file 2 — Complex Leadership Studies in Non-healthcare Settings and Reasons for Exclusion. [file ijhpm-7-1073-s002.pdf]

Supplementary file 2. Complex Leadership Studies in Non-healthcare Settings and Reasons for Exclusion

**References to Excluded Papers**	**Reason of Exclusion**
Mendes M, Mendes M, Gomes C: Promoting learning and innovation in organizations through complexity leadership theory. Team Performance 2016.	Conceptual Paper
Gilbert D, Shrieves L, Yearworth M: A Case Study of Applying Complexity Leadership Theory in Thales UK. Complex Systems Design \& 2016.	Engineering
Anderson RJ, Adams WA: Book Highlight—Mastery and Maturity, Consciousness, and Complexity: The Leadership Development Agenda. Global Business and Organizational Excellence 2016, 35(4):76-87.	Conceptual Paper
Yahaya M: Complexity of Leadership in the Context of Political Governance in Niger. In: Chaos, Complexity and Leadership 2013. ed.; 2015.	Public Administration
White BE: On Leadership in the Complex Adaptive Systems Engineering of Enterprise Transformation. Journal of Enterprise Transformation 2015.	Engineering
Scholten P, Keskitalo ECH: Bottom-up initiatives toward climate change adaptation in cases in the Netherlands and the UK: a complexity leadership perspective. Environment and Planning 2015.	Environment
Santos DVB: Complexity leadership theory and its relation to team effectiveness: explicit coordination as a key mechanism. 2015.	Public Administration
Rose EJ: A case study in complexity leadership: Preparing baccalaureate nursing students. 2015.	Education
Olalere A: Complexity and leadership crisis in Africa. International Journal of Public Leadership 2015.	Conceptual Paper

Niemandt CJP: Complex leadership as a way forward for transformational missional leadership in a denominational structure. HTS Theological Studies 2015.	Religious organisation
Muthan VM: Using chaos and complexity theory to design robust leadership architecture for South African technology businesses. 2015.	Industries
Henriquez CMG: Adaptive Leadership and Social Movements, Applying The Complex Theory of Leadership. Proceedings of the 58th 2015.	Social movement
Hazy JK, Uhl-Bien M: Towards operationalizing complexity leadership: How generative, administrative and community-building leadership practices enact organizational outcomes. Leadership 2015.	Conceptual Paper
Gantasala PV: Complexity Leadership and Knowledge Management in Higher Education. World Academy of Science and Engineering 2015.	Education
Eric G F, Yvonne R: The Challenge of Leadership Throughout the Organizational Life Cycle. In: Building Sustainably Successful Organizations, Fifth Edition. ed.: John Wiley & Sons, Inc; 2015: 305-330.	Conceptual Paper
Elkington R, Booysen L: Innovative Leadership as Enabling Function Within Organizations: A Complex Adaptive System Approach. Journal of Leadership Studies 2015, 9(3):78-80.	Conceptual Paper
Davis H: Social complexity theory for sense seeking: Unearthing leadership mindsets for unknowable and uncertain times. Emergence: Complexity and Organization 2015.	Conceptual Paper
Cunha, C. J. C. D. A., Gunther, H. F. & Gramkow, F. B. 2015b. Leadership and Complexity : A Bibliometric Study. *Business and Management Review,* 4**,** 578-590.	Bibliometric study
Chace S: The Cuban Missile Crisis: Leadership as Disturbance, Informed by History. Journal of Leadership Studies 2015, 9(2):63-68.	Law Enforcement

Carter DR, DeChurch LA, Braun MT, Contractor NS: Social network approaches to leadership: an integrative conceptual review. The Journal of applied psychology 2015, 100(3):597-622.	Conceptual Paper
Presley SP: How leaders engage in complexity leadership: Do action-logics make a difference? Fielding Graduate University2014.	Industries
Levanti G, Picone PM: The Role Of Complex Leadership In Interfirm Strategic Networks: Enabling Effect Versus Emergence. Academy of Management 2014.	Industries
Heaslip RJ: Leading Complex Endeavors. In: Managing Complex Projects and Programs. edn.: John Wiley & Sons, Inc.; 2014: 155-169.	Conceptual Paper
Guarana CL, Hernandez M: Building sense out of situational complexity The role of ambivalence in creating functional leadership processes. Organizational Psychology Review 2014.	Conceptual Paper
Gregoire MB, Arendt SW: Leadership: reflections over the past 100 years. J Acad Nutr Diet 2014, 114(5 Suppl):S10-19.	Conceptual Paper
Geer-Frazier B: Complexity leadership generates innovation, learning, and adaptation of the organization. Emergence: Complexity and Organization 2014.	Conceptual Paper
Brown BC: Sane Leadership in a Crazy World: Essentials of Complexity Leadership. Academy meta-integral org 2014.	Conceptual Paper
Van Wart M: Lessons from Leadership Theory and the Contemporary Challenges of Leaders. Public Administration Review 2013, 73(4):553-565.	Public Administration
O'Connor SJ: The complex world faced daily by health care executives and ideas and direction to effectively deal with this complexity. J Healthc Manag 2013, 58(6):387-388.	Editorial
Nooteboom SG, Termeer C: Strategies of complexity leadership in governance systems. International Review of Public 2013.	Environment

Moerschell MBAL, Banner DK, Lao T, Singer T: Complexity Change Theory: Improvisational Leadership For Complex And Chaotic Environments. Leadership & Organizational Management Journal 2013, 13(1):24.	Conceptual Paper
Mason RB: Complexity Theory and Leadership for Turbulent Environments. J Soc Sci 2013.	Industries
Lindstrom RR: Leadership needs to shift in the health system: three emerging perspectives to inform our way forward. Healthc Pap 2013, 13(1):48-54; discussion 78-82.	Editorial
Liang TY: Edge of emergence, relativistic complexity and the new leadership. Human Systems Management 2013.	Editorial
Kowch EG: Conceptualising the essential qualities of complex adaptive leadership: networks that organise. Journal of Complexity in Leadership and in Management 2013.	Conceptual Paper
Hudson DL: Attachment theory and leader-follower relationships. The Psychologist-Manager Journal 2013, 16(3):147-159.	Conceptual Paper
Hazy JK: Rethinking Complexity Leadership. In: 73rd Annual Meeting of the Academy of Management. 2013.	Conference paper
Galbraith WS: The Complex Leadership Challenges of Joint Basing. 2013.	Law Enforcement
Curlee W, Gordon RL: Complexity and Program Management: leadership and complexity. In: Successful Project Management Strategies of Complexity edn.: John Wiley & Sons, Inc.; 2013: 103-115.	Conceptual Paper
Clarke N: Model of complexity leadership development. Human Resource Development International 2013.	Conceptual Paper
Booker R: Leadership of education psychological services: fit for purpose? Educ Psychol Pract 2013, 29(2):197-208.	Psychology
Bento FC: Organizational complexity: leadership and change in research-intensive academic departments. Norwegian University of Science and Technology; 2013.	Education
Antoniadis D: Leadership Style and Socio-Organo Complexity: Managing Its Effects. Business Systems Review 2013.	Conceptual Paper

Alimo-Metcalfe B: A Critical Review of Leadership Theory. In: The Wiley-Blackwell Handbook of the Psychology of Leadership, Change, and Organizational Development. edn.: John Wiley & Sons; 2013: 13-47.	Conceptual Paper
Zenouzi BN, Dehghan A: Complexity Theory and General Model of Leadership. Global Journal of Management 2012.	Conceptual Paper
Termeer C, Nooteboom SG: Complexity leadership for sustainable regional innovations. In: Leadership and Change in Sustainable Regional Development. edn. Edited by Sotarauta M, Horlings I, Liddle J; 2012.	Conceptual Paper
Terke¸sli R: Chaos, Complexity and Police Leadership. In: Chaos, complexity and leadership edn.; 2012.	Law Enforcement
Stacey RD: Tools and techniques of leadership and management: Meeting the challenge of complexity; 2012.	Conceptual Paper
Psychogios AG, Garev S: Understanding Complexity Leadership Behaviour In SMEs: Lessons From A Turbulent Business Environment. ECO, 2012.	Industries
Olmedo E: The future of leadership: The new complex leaders' skills. Academic Research 2012.	Conceptual Paper
Neyişci N, Potas N: New Leadership Paradigms in the Complexity Science. In: Chaos, Complexity and Leadership 2012. Edited by Banerjee S, Erçetin SSu: Springer Proceedings in Complexity 2012.	Education
Morpurgo S: Leadership and Complexity. In: Projects and Complexity. edn. Edited by Francesco Varanini WG; 2012: 215-234.	Conceptual Paper
Marion R, Rumsey MG: Organizational leadership and complexity mechanisms. In: Oxford Handbook of Leadership. Ed: 2012.	Conceptual Paper
Krisko A, Stiles E: Learning to trust in organisational complexity–A theoretical framework of complexity leadership and collaborative community theories. 2012.	Conceptual Paper
Edson MC: A Complex Adaptive Systems View of Resilience in a Project Team. Systems Research and Behavioral Science 2012, 29(5):499-516.	Education

Bulutlar F, Kamaşak R: Complex Adaptive Leadership for Performance: A Theoretical Framework. In: Chaos, Complexity and Leadership. edn.; 2012.	Conceptual Paper
Brown BC, Center IS: Essentials of Applying Complexity Thinking for Sustainability Leadership. Retrieved May 2012.	Conceptual Paper
Antoniadis DN: Leadership Style and the Management of the Effects of Complexity. In: Chaos and Complexity Theory for Management: Nonlinear Dynamics. edn.; 2012.	Duplicate (Book Chapter)
Allen TW: Confronting Complexity and Creating Unity of Effort: The Leadership Challenge for Public Administrators. Public Administration Review 2012, 72(3):320-321.	Public Administration
Uhl-Bien M, Marion R, McKelvey B: Complexity leadership theory: shifting leadership from the industrial age to the knowledge era (Ch 8). In: Leadership, Gender, and Organisation. edn.; 2011.	Conceptual Paper
Ronn H: Complexity and leadership: Conceptual and competency implications. 2011.	Conceptual Paper
Robinson WT: Organizational Complexity and Leadership. 2011.	Conceptual Paper
Raghavendran S: Sensemaking of complexity: leadership in financial services. Journal of Business 2011.	Industries
Morrison K: Leadership for self-organisation: complexity theory and communicative action. Journal of Complexity in Leadership 2011.	Conceptual Paper
Marion R, Uhl-Bien M: Implications of complexity science for the study of leadership. In: The SAGE handbook of complexity and management. edn. Edited by Allen P, Maguire S, McKelvey B. Los Angeles, London, New Delhi, Singapore, Washington DC SAGE; 2011.	Conceptual Paper
Lord RG, Hannah ST, Jennings PL: A framework for understanding leadership and individual requisite complexity. Organizational Psychology 2011.	Conceptual Paper

Kilburg RR, Donohue MD: Toward a “grand unifying theory” of leadership: Implications for consulting psychology. Consulting Psychology Journal: Practice and Research 2011, 63(1):6-25.	Consulting
Hill RM: Complexity Leadership: New Conceptions for Dealing with Soldier Suicides. Military Review 2011.	Law Enforcement
Hazy JK: Parsing the 'influential increment ‘in the language of complexity: Uncovering the systemic mechanisms of leadership influence. International Journal of Complexity in Leadership 2011.	Conceptual Paper
Hannah ST, Lord RG, Pearce CL: Leadership and collective requisite complexity. Organizational Psychology 2011.	Conceptual Paper
Gibson R: Rethinking the future: rethinking business, principles, competition, control, leadership, markets and the world 2011.	Conceptual Paper
DeLia E: Complexity leadership in industrial innovation teams: a field study of leading, learning and innovating in heterogeneous teams. 2011.	Industries
Dayton DK: Communicating organizational quality: A phenomenological study through the lenses of complexity leadership and organizational learning theories. 2011.	Inaccessible thesis
Bento F: A complex perspective towards leadership in academic departments: investigating organisational changes in a Norwegian research-intensive academic department. International Journal of Complexity in Leadership 2011.	Education
Bento F: The contribution of complexity theory to the study of departmental leadership in processes of organisational change in higher education. International Journal of Complexity in Leadership 2011.	Education
Woodhill J: Capacities for Institutional Innovation: A Complexity Perspective. IDS Bulletin 2010, 41(3):47-59.	Conceptual Paper
Winkler, I: Contemporary leadership theories: Enhancing the understanding of the complexity, subjectivity and dynamic of leadership; 2010.	Conceptual Paper

Sweetman D: Exploring the adaptive function in complexity leadership theory: An examination of shared leadership and collective creativity in innovation networks. 2010.	Education
Morrison K: Complexity theory, school leadership and management: questions for theory and practice. Educational Management Administration \& 2010.	Education
McKelvey B: Complexity leadership: the secret of Jack Welch's success. Journal of Complexity in Leadership and Management 2010. vol 1 N 1 2010	Conceptual Paper
Lotrecchiano GR: Complexity leadership in transdisciplinary (TD) learning environments: A knowledge feedback loop. International journal of transdisciplinary research 2010, 5(1).	Industries
Kaiser RB, Overfield DV: Assessing flexible leadership as a mastery of opposites. Consulting Psychology Journal: Practice and Research 2010, 62(2):105-118.	Psychology
Hazy JK: Complexity Thinking & Leadership: How Nonlinear Models of Human Organizing Dynamics Can Inform Management Practice. In: Adelphi University School of Business Working. 2010.	Conceptual Paper
Hannah ST, Jennings PL, Nobel OB-Y: Tactical military leader requisite complexity: Toward a referent structure. Military Psychology 2010, 22(4):412-449.	Law Enforcement
Goldstein J, Hazy J, Lichtenstein B: Complexity and the nexus of leadership: Leveraging nonlinear science to create ecologies of innovation; 2010.	Conceptual Paper
Davidson SJ: Complex responsive processes: a new lens for leadership in twenty-first-century health care. Nurs Forum 2010, 45(2):108-117.	Conceptual Paper
Curlee W, Gordon RL: Leadership of Complexity-Driven Organizations. In: Complexity Theory and Project Management. edn.: John Wiley & Sons, Inc.; 2010: 239-254.	Conceptual Paper

Curlee W, Gordon RL: Virtual Leadership through Complexity. In: Complexity Theory and Project Management. edn.: John Wiley & Sons, Inc.; 2010: 117-134.	Conceptual Paper
Uhl-Bien M, Marion R: Complexity leadership in bureaucratic forms of organizing: A meso model. The Leadership Quarterly 2009.	Conceptual Paper
Sims BD: Complexity, adaptive leadership, phase transitions, and new emergent order: A case study of the Northwest Texas Conference of the United Methodist Church. 2009.	Religious organisation
Pfaff H, Neumann M, Kuch C, Hammer A, Janssen C, Brinkmann A, Ommen O The basic principles of leadership. Z Evid Fortbild Qual Gesundhwes 2009, 103(4):187-192.	Conceptual Paper
Nooteboom A, Termeer C: Sustainable transitions and complexity leadership. 2009.	Conceptual Paper
Newth J, Corner PD: Leadership in new ventures: complexity managed by teams. International Journal of Business 2009.	Industries
Martinez S: Emergent Leadership, Linking complexity, cognitive processes, adaptability and Innovation. Crosscutting Issues in International Transformation 2009.	Law Enforcement
Johannessen SO: The complexity turn in studies of organisations and leadership: relevance and implications. International Journal of Learning and change 2009.	Conceptual Paper
Burchell J: The Practical Application of Transformational Theory Vs. Complexity Leadership Theory on the Challenges of Leading. Journal of Business & Leadership (2005-2012) 2009.	Industries
Avolio BJ, Walumbwa FO, Weber TJ: Leadership: current theories, research, and future directions. Annu Rev Psychol 2009, 60:421-449.	Conceptual Paper
Velsor EV: A complexity perspective on leadership development. In: Complexity leadership, part 1. edn. Edited by Uhl-Bien M, Marion R; 2008.	Conceptual Paper

Uhl-Bien M, Marion R, McKelvey B: Complexity leadership theory Shifting leadership from the industrial age to the knowledge era Chapter 8 . In: Complexity leadership: part 1. edn.; 2008.	Conceptual Paper
Uhl-Bien M: Complexity leadership : Part 1. USA Information aged publishing 2008.(Book)	Conceptual Paper
Sangvai D, Lyn M, Michener L: Defining high-performance teams and physician leadership. Physician Exec 2008, 34(2):44-51.	Education
McKelvey B: Emergent strategy via complexity leadership. In: Complexity and Leadership: Part I. edn. Edited by Bien MU; 2008.	Conceptual Paper
Marion R: Complexity theory for organizations and organizational leadership. In: Complexity leadership, part 1. ed. Edited by Bien MU, Marion R. Charlotte North Carolina: Information Age Publishing; 2008.	Conceptual Paper
Lauser B, Peters M: A complexity perspective on leadership and change in the post-merger integration process. International Journal of Learning and 2008.	Industries
Jackson PR, Leach DJ, Charles K: A longitudinal study of complexity leadership in empowered work teams. Available at SSRN 1508544 2008.	Industries
Hazy JK: Toward a theory of leadership in complex systems: computational modeling explorations. Nonlinear Dynamics Psychol Life Sci 2008, 12(3):281-310.	Conceptual Paper
Hannah ST, Eggers JT, Peter LJ: Complex Adaptive Leadership: defining what constitutes effective leadership for complex organisational contexts. In: Knowledge-driven corporation: complex creative destruction. edn.; 2008.	Conceptual Paper
Dooley KJ, Lichtenstein B: Research methods for studying the complexity dynamics of leadership. In: Complexity leadrship part I:. edn. Edited by Uhl-Bien M; 2008.	Research Methodology
Clancy TR, Effken JA, Pesut D: Applications of complex systems theory in nursing education, research, and practice. Nursing outlook 2008, 56(5):248-256.e243.	Education

Boyatzis RE: Leadership development from a complexity perspective. Consulting Psychology Journal: Practice and Research 2008, 60(4):298-313.	Psychology
Uhl-Bien M, Marion R, McKelvey B: Complexity leadership theory: Shifting leadership from the industrial age to the knowledge era. The leadership quarterly 2007.	Conceptual Paper
Sorlie S: A complex task: Creating models for responsible leadership. Leadership in Action 2007, 27(1):21-23.	Industries
Simpson P: Organizing in the mist: a case study in leadership and complexity. Leadership \& Organization Development Journal 2007.	Psychology
Moody RC, Horton-Deutsch S, Pesut DJ: Appreciative inquiry for leading in complex systems: supporting the transformation of academic nursing culture. J Nurs Educ 2007, 46(7):319-324.	Education
Marion R, Uhl-Bien M: Introduction to Leadership Quarterly Special Issue on Leadership and Complexity. 2007.	Conference paper
Marion R, Uhl-Bien M: Complexity and strategic leadership. In: Monographs in leadership and management volume 4 : being there even when you are not : leading through strategy, structures and systems Edited by Hooijberg R, Hunt JGJ, Antonakis J, Boal KB, Lane N; 2007.	Conceptual Paper
Lichtenstein BB, Uhl-Bien M, Marion R, Seers A, Orton JD, Schreiber C: Complexity leadership theory: An interactive perspective on leading in complex adaptive systems. 2006.	Conceptual Paper
Kezar AJ, Carducci R, McGavin MC: Complexity and Chaos Theory. In: Rethinking the "L" Word in Higher Education: the reveolution of research on leadership Volume 31, edn.: Wiley Subscription Services, Inc., A Wiley Company; 2006: 1-218.	Education
Goldstein JA, Hazy JK: Editorial introduction to the special issue: From complexity to leadership and back to complexity. Emergence-Mahwah- 2006.	Editorial

Christensen NL: The leadership challenges of complexity and uncertainty in environmental science. Leader to Leader 2006, 2006(S1):20-23.	Environment
Souba WW: New ways of understanding and accomplishing leadership in academic medicine. J Surg Res 2004, 117(2):177-186.	Clinical practice
Marion R, Uhl-Bien M: Complexity theory and Al-Qaeda: Examining complex leadership. Emergence 2003.	Terrorism
Avolio BJ, Sosik JJ, Jung DI, Berson Y: Leadership Models, Methods, and Applications. In: Handbook of Psychology. Ed John Wiley & Sons, Inc.; 2003: 277-308.	Conceptual Paper
Poley JK: Leadership. New Directions for Higher Education 2001, 2001(115):83-93.	Education
Marion R, Uhl-Bien M: Leadership in complex organizations; 2001.	Conceptual paper
