# Supplementary material for: Complex Leadership in Healthcare: A Scoping Review
Source: Int J Health Policy Manag. 2018 Sep 1;7(12):1073–84. doi: 10.15171/ijhpm.2018.75 (PMC6358662; doi:10.15171/ijhpm.2018.75)
Supplement: Supplementary file 3 — Measurement and Interpretation of Kappa Coefficient [file ijhpm-7-1073-s003.pdf]

Supplementary file 3. Measurement and Interpretation of Kappa Coefficient

Table 1. Degree of Agreement Between Two Reviewers

	**Reviewer 2 Reject**	**Reviewer 2 Accept**	**Total**
Reviewer 1 Reject	62	4	66
Reviewer 1 Accept	5	13	18
Total	67	17	84

Table 2. Measurement of Kappa Coefficient

**(*P0*) Observed Agreement**	**(*PE*) Expected Agreement**	***K***	***se(K)***	**Lower 95% CI**		**Upper 95% CI**
0.893	0.670	**0.675**	0.102	**0.475**	**to**	**0.876**

Table 3. Interpretation of Kappa Coefficient

**Interpretation**		
**Value of *K***	**Strength of agreement**	
<0.20	Poor	
0.21 - 0.40	Fair	
0.41 - 0.60	Moderate	
0.61 - 0.80	Good	
0.81 - 1.00	Very good	
