# Supplementary material for: Complex Leadership in Healthcare: A Scoping Review
Source: Int J Health Policy Manag. 2018 Sep 1;7(12):1073–84. doi: 10.15171/ijhpm.2018.75 (PMC6358662; doi:10.15171/ijhpm.2018.75)
Supplement: Supplementary file 4 — Research Question in Complex Leadership [file ijhpm-7-1073-s004.pdf]

Supplementary file 4. Research Question in Complex Leadership

**Ref .**	**Research Aim/Objective**	**Level of Analysis**
[29] (Anderson, 2000)	"to develop new insights for management by looking at the intersection of these two views of health care organizations, the organization as a professional organization and the organization as a complex adaptive system."	Mesolevel
[30] (Burns, 2001)	"To explore whether complexity principles make sense to front-line healthcare Leaders."	Microlevel
[31](Plsek, 2001)	" To apply complexity thinking in the organization and management of health care."	Mesolevel
[32](Minas, 2005)	"To examine clinician leadership for change from the perspective of complexity."	Microlevel
[33] (Penprase, 2005)	"To provide an overview of CAS theory and its application in nursing management."	Mesolevel
[34] (Forbes Thompson, 2007)	"To describe the working conditions in four nursing homes—two high performing and two low performing—through the lens of complexity science theory."	Mesolevel
[35] (Ford,2009)	"To identify competencies of complex leadership necessary to manager new complex adaptive organisation and highlight underlying logic of how a complex adaptive organization and how complex leaders develop practices and principles in leading and managing these new emerging organizations?”	Mircolevel
[36] (Chadwick, 2010)	"To introduce the Complex Adaptive Leadership Model as a means to promote culture change and promote productive conflict."	Mesolevel

[37] (Davidson, 2010)	To discuss the use of complexity science and, specifically, complex responsive processes (CRPs) “as a helpful scaffold for leadership in twenty-first-century health care,” and suggest “the seven da Vincian principles as a personal tool that twenty-first-century leaders might use to increase their capacity for creativity and to develop their ability to thrive in uncertainty."	Microlevel
[38] (Gonnering, 2010)	To emphasize the need for physicians, to understand differences between simple complicated complex and chaotics problems using complexity tools such as Stacey diagrams and Cynefin Model.	Microlevel
[39] (Hanson, 2010)	To examine how “sections of a hospital laboratory interact within a network complexity leadership perspective and what sections display enabling leadership tendencies at a collective level."	Mesolevel
[40] (Martin, 2010)	To provide a conceptual and theoretical context on comparative complexity of family medicine consultations in the USA to provide models for adaptive leadership in clinical care and social networking to make sense of health promotion challenges for young people and to suggest "Sense-Making” schema.	Micro level
[41] (Ott, 2010)	To understand and describe the nature of the relationships and interactions among individuals, groups, and information that enables effective successful leadership of radical product innovation in the biomedical context.	Microlevel
[42] (Price, 2011)	“To emphasise the role of using ‘lens of complexity’ to ‘empowers leaders and managers in primary care to adapt their styles according to the needs of the organisation’ and offer of model for training clinical leaders."	Micro level

[43] (Bailey, 2012)	"To describe the Adaptive Leadership framework and how it can be used to explore health care provider leadership behaviors at the point of care and demonstrate the application of the Adaptive Leadership framework to nursing research."	Mircolevel
[44](MacCarthey, 2012)	To establish the extent to which Organisational Complexity moderates Behavioural Complexity and leadership effectiveness and the extent to which leadership training supports Behavioural Complexity in contributing to leadership effectiveness.	Meso & Microlevel
[45](Sturmberg, 2012)	“To stress the need for rigorous scientific application of complexity science in healthcare and bring a good relevant reference of application of complexity in health."	Mesolevel
[46] (Weberg, 2012)	To present the contrasting option of complexity leadership, in comparison with traditional leadership models, and its relationship with improved outcomes in health care.	Mesolevel
[47] (Corazzini, 2013)	To describe examples of adaptive leadership to facilitate assessment, care planning, supervision and delegation.	Microlevel
[48](Lindstrom, 2013)	To understand (using complexity leadership, neuroleadership and phronetic leadership) the underlying factors that help or hinder the kind of leadership that engages multiples stakeholders, front line leadership in service of front line ownership.	Microlevel :
[49](Weberg, 2013)	“This study will use the complexity leadership theory (CLT) lens to examine complexity leadership in regard to innovation in simulation centre context."	Mesolevel
[50](Cohn, 2014)	Contrast leadership in complexity from traditional leadership approaches and stresses the need for an adaptive space leadership and give illustration from an intensive care unit and a chaplaincy service.	Microlevel
[51](Gilson, 2014)	“Considers how actors’ sense making and the exercise of discretionary power currently combine to challenge primary health care (PHC) reorientation in the South African health	Mesolevel

	system and provides examples of leadership practices that promote sense making and power use in support of PHC."	
[52](Prashanth, 2014)	"To describe the complexity of a capacity-building intervention at the district level and illustrate the utility of the realist approach in advancing the practice of systems thinking in complex settings."	Mesolevel
[53](Viitala, 2014)	“To improve understanding of how leaders themselves construct leadership in relation to organisational change." This paper presents a longitudinal study of leadership transformation in the nursing function of one hospital.	Meso level
[54](Anderson, 2015)	We propose our framework, derived from Heifetz et al’s Adaptive Leadership Framework, as a guide to the study of trajectories of changes in chronic illness symptoms and for developing better tools for providing patient-centred care.	Micro level
[55](Crowell, 2015)	"This book provides a framework for complexity nursing leadership rooted in complexity science. It reflects a three-part emphasis on knowledge, leadership style, and personal being and awareness"	Micro level
[56](Grady,2015)	"To examine physician leadership development using principles of complexity science. It explores whether physician leadership development within health-care organizations facilitate improved outcomes by examining complex system behaviours?"	Micro level
[57](Kwamie, 2015)	To understand systemic influences on decision-making and the type of leadership likely to emerge out of these systemic interactions within district health management teams with regard to implementation of maternal and newborn health policies.	Mesolevel & Microlevel

[58](Linderman, 2015)	"To examine a methodology for capturing and sharing the wisdom of experienced nursing leaders and suggest the application of principles of sense making in improving the leader's own leadership capacity, while enhancing that of their colleagues and subordinates"	Microlevel
[59](Mckimm, 2015)	This article explores how an understanding of approaches to leading and managing change and complexity science can help clinical leaders engage with and manage change in complex environments and systems more effectively.	Microlevel
[60](Porter-O-Grady, 2015)	To explore basic principles of complexity, their impact on teams, and the fundamental elements of team effectiveness.	Microlevel
[61] (Prescott, 2015)	To review some of the principal debates surrounding the ideas of organizational culture, leadership and change as they relate to the NHS, to make explicit the complex and contested nature of these ideas.	Macrolevel
[62](Arena, 2016)	Advocates that "professionals need to more strongly consider social capital strategies in driving both performance and innovation within complex organizations."	Mesolevel
[63](Howard, 2016)	" to set the topic of Innovation firmly within the context of leadership and show how both collective leadership paradigms and individual leadership styles influence innovation within the pharmaceutical industry."	Microlevel
[64](Miller, 2016)	The purpose of this paper is to investigate adaptive language constructs for consumers within the complex system of behavioural healthcare."	Individual
[65](Weberg, 2016)	describes:<br>1) how leadership emerges from the interactions of team members to move innovation forward in an organization.<br>2) the inadequacies of traditional leadership practices in addressing innovation behaviours	Microlevel

	3) seven behaviours of innovation and how leaders can recognize and apply them to their practice.	
