# Supplementary material for: Complex Leadership in Healthcare: A Scoping Review
Source: Int J Health Policy Manag. 2018 Sep 1;7(12):1073–84. doi: 10.15171/ijhpm.2018.75 (PMC6358662; doi:10.15171/ijhpm.2018.75)
Supplement: Supplementary file 5 — Scope, Epistemology, Theory and Conceptual Definitions of Complex Leadership. [file ijhpm-7-1073-s005.pdf]

**Supplementary file 5.** Scope, Epistemology, Theory and Conceptual Definitions of Complex Leadership

| **Author, date** | **Conceptual definitions** | **Theory** | **Epistemolog y** | **Universal versus situational** | **Scope of leadership** |
|---|---|---|---|---|---|
| [29] (Anderson, 2000) | The task of managing a professional complex adaptive system is not to know what is going on and then tell others in the organization what to do. But the task is to create a learning organization. | CAS[1] | Rule based | Universal | Comprehensive |
| [30] (Burns, 2001) | Leadership that uses complexity principles offers opportunities in the chaotic healthcare environment to focus less on prediction and control and more on fostering relationships and creating conditions in which complex adaptive systems can evolve to produce creative outcomes. | CAS | Rule based | Situational | Comprehensive |
| [31](Plsek,2001) | "…effective organisation and delivery of health care does not need detailed targets and specifications, nor should it focus primarily on 'controlling the process' or 'overcoming resistance.' Rather, those who seek to change an organisation should harness the natural creativity and organising ability of its staff and stakeholders through such principles as generative relationships, minimum specification, the positive use of attractors for change, and a constructive approach to variation in areas of practice where there is only moderate certainty and agreement." | CAS | Rule based | Situational | Narrow (enabling leadership) |

[1] Complex Adaptive Systems

| | | | | | |
|---|---|---|---|---|---|
| [32] (Minas, 2005) | Complex leadership is about "setting minimum specifications that allow progress towards goals, creativity, and innovation,.., Understanding attraction for change rather than battling resistance, learning for capability, clear and explicit articulation of the values that will underpin everything else that occurs in the system." | CAS | Rule based | Situational | Narrow (Adaptive leadership) |
| [33] (Penprase, 2005) | "Short term goal setting and creating clear vision that enable organization to be flexible and adaptive, it is to decipher trends assist staff to accept and adjust to change, support coordination of differents element of change and help the nursing department to maintain its focus and identify as it moves through a turbulent health care management" | CAS | Rule based | Universal | Narrow(enabling leadership) |
| [34](Forbes Thompson, 2007) | 1) Creating a positive, coherent, and explicit organizational mission/identity, one that is consistent with fundamental human values, will serve as an attractor to foster trust and staff connectedness. 2) Relationships and self-organization of team in positive and creative ways to get the work done". 3) Fewer, more flexible rules, when grounded in trusting relationships and a clear mission, will empower staff and allow greater creativity in meeting resident needs. | CAS | Rule based | Universal | Narrow (enabling leadership) |
| [35] (Ford, 2009) | "Leader effectiveness depends on the ability to foster conditions that allow for a productive future to emerge." Three fundamental activities that enable managing turbulence in a non-equilibrium environment: (1) how to foster network construction at the frontline, middle and top of the organization; (2) how to plant seeds to catalyse emergence from the bottom-up; (identify knowledge centres within the organization and encourage these centres to communicate with one another and engage in creative problem-solving, and (3) how to nurture systemic thinking. | CAS | Rule based | Situational | Narrow (enabling leadership) |

| | | | | | |
|---|---|---|---|---|---|
| [36](Chadwick, 2010) | Complex adaptive leadership framework (leadership-team-culture) Leadership is participative, described as the ability to accept comfortable and uncomfortable situations, to facilitate dialogue between various levels of staff, let go the desire of control; and to grasp the on-going challenges in health care as well as energize those they serve (servant leadership). | Quantum theory & CAS | Rule based | Universal | Comprehensive |
| [37](Davidson, 2010) | Leadership and the creation of meaning arise from the living present, and the living present is a process of participation and self-organization between people. Leaders, then, are those among us who seem to have a heightened capacity for facilitating transformation and whose participation in the living present is characterized as highly novel and creative | Complex responsive process | Heuristic Based ontology | Situational | Comprehensive |
| (Gonnering, 2010) | "The physician executive must actively foster the development of an organizational culture capable of operating at the edge of chaos, maximizing the creation of value for all stakeholders while at the same time retaining the resilience and nimbleness to recognize changes in opportunity." Blue ocean of opportunity | CAS | Rule based | situational | Comprehensive |
| [39](Hanson, 2010) | CLT examines leadership as a process involving networks of highly interactive, interdependent members leading to collaboration, creativity, innovation, and other outcomes needed for organizational adaptation. Complexity leadership incorporates three types of leadership functions: adaptive, enabling, and administrative (Uhl-Bien et al, 2007). "From a complexity perspective, there exist both positional and informal leaders fulfilling diverse functions. Formal leaders carry the authority of position; informal leaders emerge based on relationship" | CAS | Rule based | Universal | Comprehensive |
| [40] (Martin, 2010) | "Adaptive leadership by definition straddles the polarities, tensions and contradictions of the clinical encounter … The Complex Network Electronic | Multi- | Heuristic | Universal | Comprehensive |

| | | | | | |
|---|---|---|---|---|---|
| | Knowledge Translation Research Model represents an attempt to address these issues using a social organizing approach based on complexity science, social learning theories, design thinking, and models of knowledge exchange, translation and integration." | Ontology sense-making2 | | | |
| [41] (Ott, 2010) | The author adopts the complexity leadership theory (1) adaptive leadership (process creation) 2) administrative leadership (Maintenance issues or management) 3) and enabling leadership (creating a context appropriate for rapid adaptation. "Leadership occurs in interaction between agents to allow for adaptation, creativity, and learning" | CAS (CLT)[3]. | Rule based | Universal | Comprehensive |
| **[42]** (Price, 2011) | Leadership is about moving out of the 'safe' zone of stability and order into the 'zone of complexity,' where creativity and change can occur. | Edgeware[4] | Heuristic | Situational | Not clear |
| [43] (Bailey, 2012) | "Leadership emerges in day to day work as people interact with each other to do their jobs. Adaptive leadership is the work that practitioners do to mobilize and support patients to do the adaptive work. Adaptive leadership is fundamentally a non-linear, iterative, reciprocal interaction between the health care practitioner and the patient" | CAS | Rule based | Situational | Narrow (Adaptive leadership) |
| [44] (MacCarthey, 2012) | "Complex Leaders possess a strong sense of Cognitive, Social, and Behavioural Complexity (leaderplex model) that contribute to their effectiveness" | Cognitive, social behavioural complexity | Heuristic | Situational | Comprehensive |

[2] Mathematical modeling, communication, organisational studies)
[3] Complexity Leadership Theory
[4] Edgeware: Complexity Diagram Stacey Zimmerman simple, complicated, complex, chaos

| | | | | | |
|---|---|---|---|---|---|
| [45](Sturmberg, 2012) | Leadership entails the skills of showing the way 'no clear definition of complexity leadership is provided’ | CAS & Chaos theory | Rule based | Universal | Unspecified |
| [46] (Weberg,2012) | "Leadership is a process that occurs in situations where groups need to learn their way out of unpredictable problems and situations."<br>“The behavior of leadership is to foster interaction from within the organization, increase network strength of the organization, and inject conflict to bust stability, creating the parameters and energy for constant change and adaptation.” | CAS | Rule based | Universal | Comprehensive |
| [47] (Corazzini, 2013) | Adaptive leadership requires having the attitudes and skills necessary to distinguish between technical and adaptive challenges and appropriately aligning the solutions, using expertise where appropriate for the technical aspects and using adaptive approaches where necessary | CAS | Rule based | Situational | Narrow (Adaptive leadership) |
| [48] (Lindstrom, 2013) | “Front line ownership or distributed leadership as context specific, multiple stakeholders perspective and multilevel participation"<br>complexity leadership assimilated "as the complex interplay of power, interrelationship, attentiveness, mindfulness, approach and effort, all of which were addressed to advance patients safety initiatives." | CAS & Neuro & Phronetic leadership | Heuristic Based ontology | Situational | focuses on front line leadership |
| [49](Weberg, 2013) | “Complexity leadership behaviours such as administrative, adaptive, and emergent leadership evolve together in patterns that single point-in-time studies cannot uncover. leadership recognition of interrelationships, emergence, and fostering innovation." | CAS | Rule based | Universal | Comprehensive |

| | | | | | |
|---|---|---|---|---|---|
| [50](Cohn, 2014) | "Leading in complexity requires leaders to accept the complexity, create an adaptive space in which innovation and creativity can flourish and then integrate the successful practice that emerge into the formal organizational structure." "The role of leaders in complexity includes enabling conditions that foster the potential for the system to adapt productively as well as the use of appropriate constraints help institutionalize productive principles and behaviours." | CAS | Rule based | Situational | Narrow ( adaptive space leadership) |
| [51](Gilson, 2014) | This (Primary health care reform in South Africa) requires a leadership of sensemaking that enables front line staff to exercise their collective discretionary power in strengthening PHC. "Leaders must foster learning and values... They need a sense of optimism that can help the system deal with complexity, risk taking, and uncertainty. They need to help the system maintain a coherent identity | CAS, system thinking, street level bureaucracy | Heuristic Based ontology | Universal | narrow ( Front line leadership or distributed leadership) |
| [52](Prashanth, 2014) | There were no direct explicit description of complex leadership though the authors explained how change occurs in talukas (subunit of health district in India) leadership is viewed as removing barriers to change which correspond to that enabling leadership function of Uhl-bien. | Social Construction | Heuristic | Universal | narrow ( Front line leadership |
| [53](Viitala, 2014) | "Leadership is seen here as a socially constructed product, which is at the same time institutionalised both in organisations and in a society and also continually being reproduced in everyday situations in communities. (…) The core of the issue is communication, influence and interaction between people and in this process both power and resistance play important role." | Social construction , institutionalisation theory, | Heuristic | situational | Comprehensive |

| | | | | | |
|---|---|---|---|---|---|
| [54](Anderson,2015) | "The aim of adaptive leadership is to facilitate the patient/family to do the work necessary to close the gap created by adaptive challenges by encompassing a holistic approach to patients within their life contexts" | CAS | Rule based | Situational | Narrow (adaptive leadership) |
| [55](Crowell, 2015) | Complexity leadership framework: "It reflects a three-part emphasis on knowledge, leadership style, and personal being and awareness." These components result in the fourth component of the model: actions that can best benefit the organization as a whole and the people within it. | CAS | Rule based | Situational | Comprehensive |
| [56](Grady, 2015) | "Leadership as behaviour that is non-linear, it is collective style of leadership that focuses on mutual accountability for patient outcome" | CAS historicity, interpretation, learning organization | Heuristic | Universal | Focused on physician relationship with patients and families |
| [57](Kwamie, 2015) | "Managers create enabling conditions for constructive but largely unknown future states." "Administrative leadership is largely driven by top-down structures and pays most attention to bureaucracy, hierarchy, control, and alignment; managerial roles are focused on formal planning and coordination actions to achieve organizational goals. Adaptive leadership is focused on creative actions that promote learning and innovation, often linked to bottom-up generative acts. Enabling leadership attempts to bridge administrative and adaptive functions, allowing them to work in concert rather than in opposition." | CAS theory, complexity leadership theory | Rule based | Universal | Focused (enabling leadership) |

| | | | | | |
|---|---|---|---|---|---|
| [58](Linderman, 2015) | "The concept of generativity relates to people who can see possibilities, who are open to exploring new ways of doing things, and who are continuously focused on making things better." The generative leader's expertise can be observed through illumination or and reflection upon sense-making gap. | Sense making generative & wisdom leadership | Heuristic | Situational | Narrow on front line leadership |
| [59] (Mckimm, 2015) | Adaptive leaders need to have the skills to evaluate contexts so as to create the conditions in which the organization (and those who work within it) can cope with change and develop resilience. The authors adopt Heifetz definition of adaptive leadership that 'acknowledges that leaders work within systems where inherent challenges and political dimensions are faced both internally and externally. Adaptive leaders recognize this and are able to create a culture enabling both the organization and the individuals within it to remain responsive and resilient to them in order to 'thrive. | CAS | Rule based | Universal | narrow (Adaptive leadership) |
| (Porter-O'Grady, 2015) | Use of CAS principles to team level leadership the leadership of team (emergence, self-organizing, coevolution, requisite variety, connectivity, iteration, simple rules, living on the edge of chaos, suboptimal behavior, and nested systems.) | CAS | Rule based espistemolo gy | Universal | focuses of team level dynamics |
| [61] (Prescott, 2015) | "There is need for leadership at all levels and in all professions in the complex worlds of NHS institutions. Complex leadership is believed to be distributed based on inclusivity and with the patient at the centre of decision-making processes." | CAS | Rule based | Universal | narrow ( Front line leadership or distributed leadership) |
| [62](Arena, 2016) | The authors adopt the complexity leadership theory (Uhl Bien 2007) adaptive, enabling and administrative leadership. They advocates the need for paradigm | CAS | Rule based | Universal | Comprehensive |

| | | | | | |
|---|---|---|---|---|---|
| | shift from human capital to social capital that focus more on facilitating "the movement of ideas across a system through bridging and brokering." | | | | |
| [63](Howard,2016) | Authors refers to complex leadership as "evolutionary leadership" that is “mostly about connecting with purpose, establishing (and continuously reestablishing) context, setting up the minimum key principles and processes for an Evolutionary paradigm to work, and then letting people get on with it, with buckets of trust, transparency, openness, and authenticity." | CAS | Rule based | Universal | Narrow leader follower perspective |
| [64](Miller,2016) | Leadership emerges in relationship to the needs of those who live in complex circumstances, whether or not advocacy or representation takes this into consideration. Emphasis in multi-stakeholder perspective. | "A CAS social interaction perspective | Heuristic | Universal | Comprehensive |
| [65](Weberg, 2016) | Complexity leadership theory (CLT) include leadership recognition of interrelationships, emergence, and fostering innovation, ‘multilevel leadership (administrative, enabling, adaptive)’ a complex interaction of leadership behaviours by multiple individuals in response to emergent opportunities in the internal and the external environments. (not a single individual characteristics) that lead to change adaptation and innovation. | CAS | Rule based | Universal | Comprehensive |
