# Supplementary material for: Complex Leadership in Healthcare: A Scoping Review
Source: Int J Health Policy Manag. 2018 Sep 1;7(12):1073–84. doi: 10.15171/ijhpm.2018.75 (PMC6358662; doi:10.15171/ijhpm.2018.75)
Supplement: Supplementary file 6 — Categorization Criteria of Included Papers. [file ijhpm-7-1073-s006.pdf]

Supplementary file 6. Categorization Criteria of Included Papers

Author	CAS	Situational	Comprehensive
(Anderson and McDaniel, 2000)	1	0	1
(Burns, 2001)	1	1	1
(Plsek and Wilson, 2001b)	1	1	0
(Minas, 2005)	1	1	0
(Penprase and Norris, 2005)	1	0	0
(Forbes-Thompson et al., 2007)	1	0	0
(Ford, 2009)	1	1	0
(Chadwick, 2010)	1	0	1
(Davidson, 2010)	0	1	1
(Gonnering, 2010)	1	1	1
(Hanson and Ford, 2010)	1	0	1
(Martin, 2010)	0	0	1
(Ott, 2010)	1	0	1
(Price, 2011)	0	1	0
(Bailey et al., 2012)	1	1	0
(McCarthy, 2012)	0	1	1
(Sturmberg and Martin, 2012)	1	0	0
(Weberg, 2012)	1	1	1
(Corazzini et al., 2013)	1	1	0
(Lindstrom, 2013)	1	1	0
(Weberg, 2013)	1	0	1
(Cohn, 2014)	1	1	0
(Gilson et al., 2014)	1	0	0
(Prashanth et al., 2014)	0	0	0
(Viitala, 2014)	0	1	1
(Anderson et al., 2015)	1	1	0
(Crowell, 2015)	1	1	1
Grady 2015 (Grady, 2015)	1	0	0
(Kwamie et al., 2015)	1	0	0
(Linderman et al., 2015)	1	1	0
(McKimm and Till, 2015)	1	0	0
(Porter-O'Grady, 2015)	1	0	0

(Prescott and Rowe, 2015)	1	0	0
(Arena and Uhl-Bien, 2016)	1	0	1
(Howard, 2016)	1	0	0
(Miller, 2016)	1	0	1
(Weberg, 2016)	1	0	1
